# Soluble Interleukin-2 Receptor Predicts Treatment Outcome in Patients With Autoimmune Tubulointerstitial Nephritis. A Preliminary Study

**DOI:** 10.3389/fmed.2022.827388

**Published:** 2022-02-25

**Authors:** Satoka Shiratori-Aso, Daigo Nakazawa, Saori Nishio, Yusho Ueda, Mina Eguchi, Ai Yokoyama, Junpei Yoshikawa, Takashi Kudo, Kanako Watanabe-Kusunoki, Sayo Takeda-Otera, Junya Yamamoto, Naoko Matsuoka, Nobuharu Kaneshima, Fumihiko Hattanda, Sari Iwasaki, Takahiro Tsuji, Yuichiro Fukasawa, Tatsuya Atsumi

**Affiliations:** ^1^Department of Rheumatology, Endocrinology and Nephrology, Faculty of Medicine and Graduate School of Medicine, Hokkaido University, Sapporo, Japan; ^2^Department of Nephrology, NTT East Japan Sapporo Hospital, Sapporo, Japan; ^3^Department of Pathology, Sapporo City General Hospital, Sapporo, Japan

**Keywords:** tubulointerstitial nephritis, soluble interleukin-2 receptor, autoimmune disease, therapeutic response, biomarkers

## Abstract

**Background:**

Autoimmune tubulointerstitial nephritis (TIN) is characterized by immune-mediated tubular injury and requires immunosuppressive therapy. However, diagnosing TIN and assessing therapeutic response are challenging for clinicians due to the lack of useful biomarkers. Pathologically, CD4^+^ T cells infiltrate to renal tubulointerstitium, and soluble interleukin-2 receptor (sIL-2R) has been widely known as a serological marker of activated T cell. Here, we explored the usefulness of serum sIL-2R to predict the treatment outcome in patients with autoimmune TIN.

**Methods:**

Study Design: Single-center retrospective observational study.

**Participants:**

62 patients were diagnosed of TIN from 2005 to April 2018 at Hokkaido University Hospital. Among them, 30 patients were diagnosed with autoimmune TIN and treated with corticosteroids. We analyzed the association between baseline characteristics including sIL-2R and the change of estimated glomerular filtration rate (eGFR) after initiation of corticosteroids.

**Results:**

The serum sIL-2R level in patients with autoimmune TIN was significantly higher than that in chronic kidney disease patients with other causes. Mean eGFR in autoimmune TIN patients treated with corticosteroids increased from 43.3 ± 20.4 mL/min/1.73 m^2^ (baseline) to 50.7 ± 19.9 mL/min/1.73 m^2^ (3 months) (ΔeGFR; 22.8 ± 26.0%). Multivariate analysis revealed that higher sIL-2R (per 100 U/mL, β = 1.102, *P* < 0.001) level was independently associated with the renal recovery. In ROC analysis, sIL-2R had the best area under the curve value (0.805) and the cutoff point was 1182 U/mL (sensitivity = 0.90, 1-specificity = 0.45).

**Conclusions:**

Our study showed that elevated serum sIL-2R levels might become a potential predictive marker for therapeutic response in autoimmune TIN.

## Introduction

Autoimmune tubulointerstitial nephritis (TIN) is characterized by immune-mediated tubular injury, recognized as either one of the organ manifestations in systemic autoimmune/autoinflammatory diseases or idiopathic TIN. Pathological studies have revealed massive CD4^+^ T cell infiltration in the renal tubulointerstitium (1), presumably responsible for the disease development.

As autoimmune TIN leads to irreversible tubular fibrosis and ultimately renal insufficiency in some of affected patients, corticosteroids and/or immunosuppressants are generally used when autoimmune TIN is diagnosed. However, immunosuppressive treatment protocol varies in the daily clinical practice (2–4), partly dependent on the underling autoimmune disease, and the standardization of the treatment of autoimmune TIN has not been established.

One of the facts of difficulty in the management of the patients with autoimmune TIN is related with the lack of specific disease biomarker. Considering the pathophysiologic involvement of CD4^+^ cells in TIN, serum levels of soluble interleukin-2 receptor (sIL-2R) (5) has been one of the candidates of the biomarker of autoimmune TIN. The determination of sIL-2R might give us some clinical information for the better management of autoimmune TIN. We therefore investigated the serum sIL-2R levels and compared their behavior with clinical parameters including treatment response using our patients' record.

## Materials and Methods

### Study Population

We retrospectively recruited patients who were admitted to Hokkaido University Hospital with a diagnosis of TIN between January 2005 and April 2018. Patients with autoimmune TIN, including idiopathic, sarcoidosis, TIN with uveitis (TINU), and immunoglobulin (Ig) G4-related kidney disease (IgG4-RKD), were enrolled in this study. The exclusion criteria were as follows: drug-induced or infectious TIN, serum creatinine ≥ 5 mg/dL, observation period <6months, and lack of sIL-2R measurements. As a control group of chronic kidney disease (CKD), we enrolled patients with IgA nephropathy (IgAN) or diabetic kidney disease (DKD), who had renal dysfunction of CKD stage 2–4, and did not have a corticosteroid therapeutic history within 6 months. This study was approved by the institutional review board of Hokkaido University Hospital (021-0113) and was conducted in accordance with the Declaration of Helsinki.

### Evaluation of Clinical and Laboratory Data

The records of all patients were reviewed for the following information: age, sex, causes of TIN, clinical manifestations, white blood cell (WBC) count, red blood cell count, hemoglobin, serum creatinine, blood urea nitrogen, albumin, C-reactive protein, venous blood gas (pH and bicarbonate), sIL-2R, IgG, complement 3, urinary protein, hematuria, urinary beta2-microglobulin (β2MG), urinary N-acetyl-beta-D-glucosaminidase (NAG), urinary sediment WBC, and initial dose of corticosteroids. The normal ranges of serum sIL-2R levels in our institution was <459U/mL. Laboratory data and clinical findings at the beginning of corticosteroid therapy or at the time of renal biopsy (in cases without treatment) were recorded as baseline data.

Estimated glomerular filtration rate (eGFR) was calculated using the following formula (6): eGFR (mL/min per 1.73 m^2^) = 194 × Cr^−1.094^ × age^−0.287^ (if female, ×0.739).

### Pathologic Studies

Pathologists at Sapporo City General Hospital performed the pathological diagnosis based on microscopic findings. Renal fibrosis was evaluated using Elastica-Masson staining, and the fibrotic area was quantified using Image J software. Interstitial inflammation was evaluated by assessing the percentage of inflammatory cell infiltration in tubulointerstitial lesions on staining with hematoxylin-eosin.

### Outcomes

The primary outcome was the recovery of renal function, defined as the change rate of eGFR from baseline to 3 months (ΔeGFR).

### Statistical Analysis

Results are expressed as numbers (percentages) for categorical data and as mean ± standard deviation or median (interquartile range) for continuous variables. Results were compared using the two-sample *t-*test or Wilcoxon rank-sum test for continuous variables. Spearman's rank correlation coefficient was used to evaluate the correlations between continuous variables. The association between biomarkers and the therapeutic response in TIN was also assessed using univariate and multivariate linear regressions in TIN patients treated with corticosteroids. The therapeutic response was defined as renal recovery (defined as ΔeGFR). A maximum of three variables for multivariate regression analysis were selected by forward selection with *P* < 0.20. Receiver operating characteristic curve analysis was performed to evaluate the predictability of improvement of renal function, which was defined as a ≥30% increase in eGFR. The area under the curve (AUC) and cutoff values were calculated. The patients were divided into two groups according to the cutoff value, and the cumulative incidence was described and compared using the log-rank test. Statistical significance was defined as *P* < 0.05. Statistical analysis was performed using JMP Pro, version 14 (SAS Institute Inc.) and GraphPad Prism version 8.0.2 for Windows (GraphPad Software).

## Results

### Patient Characteristics

**Figure 1:**
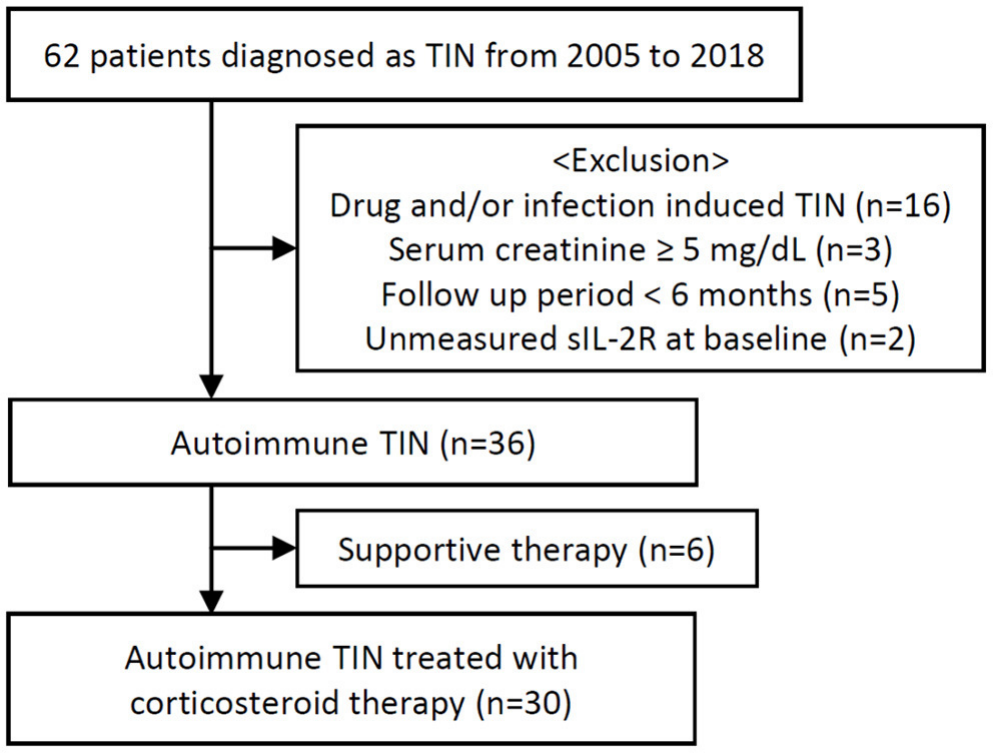
Patient flowchart.

We enrolled 62 patients with TIN between January 2005 and April 2018 at Hokkaido University Hospital. We excluded 16 patients with drug-induced TIN, 3 patients with serum creatinine ≥5 mg/dL, 5 patients with an observation period of <6 months, and 2 patients without sIL-2R measurements. Among the remaining 36 autoimmune TIN patients, 30 received corticosteroid therapy with or without immunosuppressants and 6 were not treated with neither corticosteroids nor any immunosuppressants (Figure 1).

**Figure 2:**
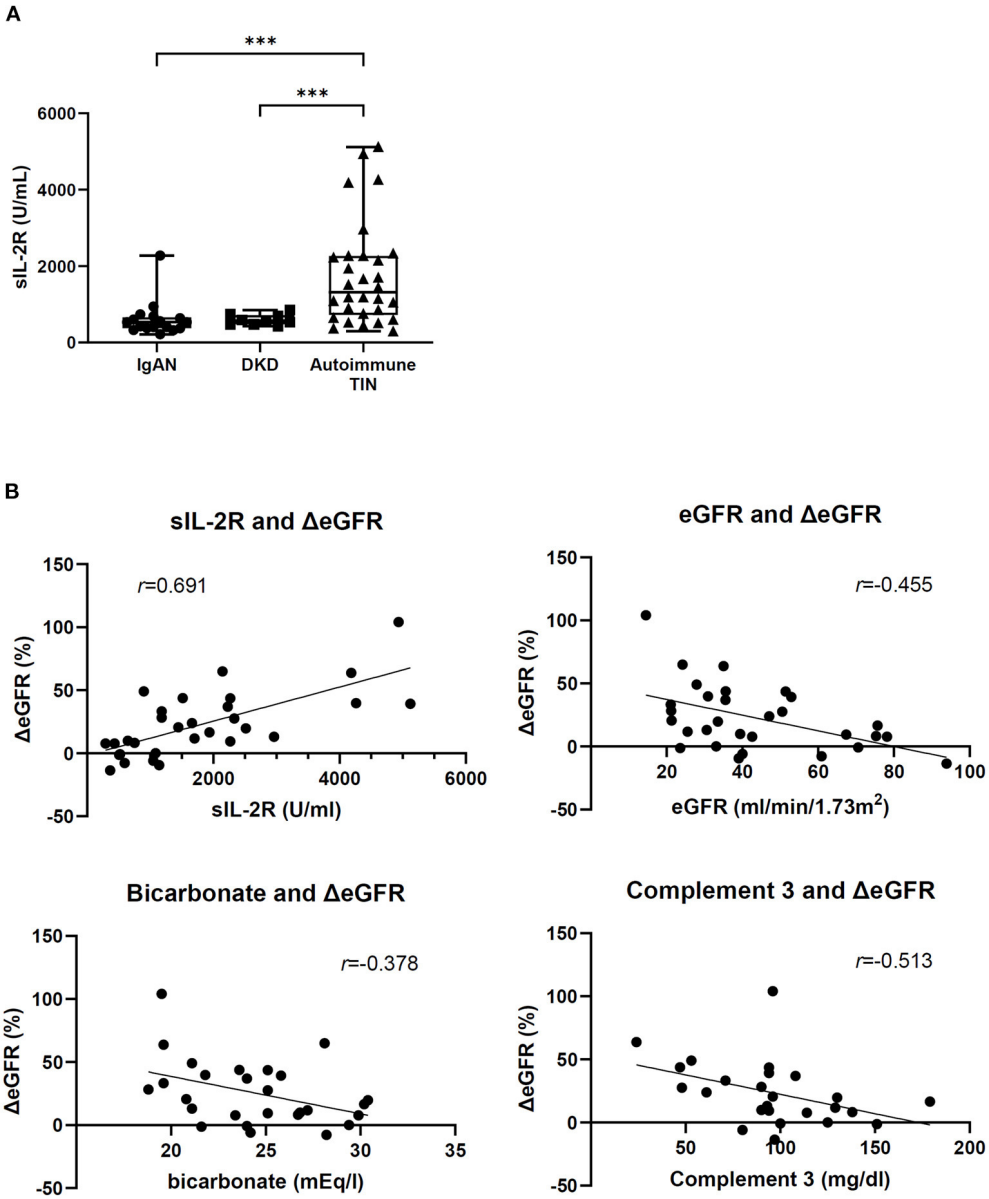
Baseline serum sIL-2R level and correlation between ΔeGFR and baseline factors. **(A)** sIL-2R level in patients with IgA nephropathy (*n* = 17), diabetic nephropathy (*n* = 9), and autoimmune TIN (*n* = 30). ***P < 0.001, Wilcoxon rank-sum test. **(B)** Correlation between ΔeGFR and baseline factors in patients with autoimmune TIN. Spearman's rank correlation coefficient (*r*) and linear regression line between ΔeGFR and baseline factors.

Supplementary Table 1 shows the patients' characteristics at baseline. The corticosteroid group comprised sarcoidosis (*n* = 10), TINU (*n* = 8), IgG4-RKD (*n* = 6), idiopathic TIN (*n* = 4), Sjögren's syndrome (*n* = 1), and Castleman disease (*n* = 1). Azathioprine was added in one patient with IgG4-RKD 15 months after the induction of corticosteroids, and tocilizumab was added in the patients with Castleman disease next month of the induction corticosteroids. Patients on corticosteroids had a median serum creatinine level of 1.31 (1.06–1.74) mg/dL and mean eGFR of 43.3 ± 20.4 mL/min/1.73 m^2^. The median sIL-2R level at baseline was 1,480 (720–2,280) U/mL. Among the biopsied 27 patients in corticosteroid group, 17 patients had available pathological data. The mean proportion of fibrotic area and median inflammatory cell infiltration was 37.3 ± 11.4 and 21.7% (17.7–47.1%), respectively. Next, because the lever of sIL-2R is known to be influenced by renal function (7), we evaluated the sIL-2R of other cause-CKD patients with renal dysfunction. Mehta et al. (8) reported that the serum sIL-2R levels of healthy volunteers was 349 ± 185 U/mL, and that of CKD patients in our study was 532 (402–689) U/mL. Patients with autoimmune TIN showed significantly a higher sIL-2R level compared to CKD patients with other cause (IgAN or DKD) (Supplementary Table 2 and Figure 2A). No significant difference in baseline eGFR was observed between these groups.

### Association Between Baseline Variables and Renal Recovery After Corticosteroid Therapy

In 30 patients treated with corticosteroids, mean eGFR increased from 43.3 ± 20.4 mL/min/1.73 m^2^ (baseline) to 50.7 ± 19.9 mL/min/1.73 m^2^ (3 months) (ΔeGFR; 22.8 ± 26.0%). In univariate analysis, high sIL-2R (per 100 U/mL, β = 1.353, *P* < 0.001), low eGFR (β = −0.621, *P* = 0.006), low bicarbonate (β = −2.967, *P* = 0.033) and low complement 3 (β = −0.307, *P* = 0.035) at baseline correlated with increasing ΔeGFR (Figure 2B). Other factors including histological parameters were not significantly associated with ΔeGFR. In multivariate analysis, high sIL-2R was an independent relative factor for increasing ΔeGFR (per 100 U/mL, β = 1.102, *P* < 0.001) (Table 1). Any other factors including age, CRP, eGFR, complement 3, and histological findings, were not significantly associated with sIL-2R levels at baseline (Supplementary Table 3).

**Table 1:** Regression analysis of renal recovery [ΔeGFR (%)] in autoimmune TIN patients.

	**Univariate**		**Multivariate**	
**Variables**	**Regression coefficient**	***P*-value**	**Regression coefficient**	***P*-value**
Age (year)	0.139	0.564		
Female	−7.118	0.463		
BMI (kg/m^2^)	1.020	0.565		
**Blood tests**				
White blood cells^$^ (100/μL)	−0.209	0.338	−0.199	0.203
Eosinophils (/μL)	0.026	0.194		
Hemoglobin (g/dL)	−3.939	0.263		
Platelets (10^4^/μL)	−1.350	0.163		
sIL-2R^#^ (100 U/mL)	1.353	<0.001^***^	1.102	<0.001^***^
Albumin (g/dL)	−6.983	0.300		
CRP (mg/dL)	−0.449	0.739		
Bicarbonate^$^ (mEq/L)	−2.967	0.033^*^	−2.054	0.059
eGFR (mL/min/1.73 m^2^)	−0.621	0.006^**^		
Immunoglobulin G (100 mg/dL)	0.552	0.159		
Complement 3 (mg/dL)	−0.307	0.035^*^		
**Urinalysis**				
Urinary protein (g/gCre)	9.316	0.124		
Hematuria	−4.353	0.705		
Leukocyturia	14.72	0.221		
β2MG/Cre (mg/gCre)	−0.036	0.721		
NAG/Cre (U/gCre)	0.069	0.676		
**Treatment**				
Initial corticosteroid dose (0.1 mg/kg)	0.838	0.708		
**Pathology**				
Inflammatory cells infiltration to interstitium (%)	−0.193	0.573		
Fibrosis (%)	−0.306	0.646		

# *Variables forced to be included in multivariate analysis*.

$ *Variables selected for multivariate analysis by forward selection with P < 0.20*.

* *P < 0.05,*

** *P < 0.01,*

*** *P < 0.001*.

*BMI, body mass index; sIL-2R, soluble interleukin-2 receptor; CRP, C-reactive protein; eGFR, estimated glomerular filtration rate; Cre, creatinine; β2MG, beta2-microglobulin; NAG, N-acetyl-beta-D-glucosaminidase; TIN, tubulointerstitial nephritis*.

### Predictive Value of sIL-2R Level for Corticosteroid Response

**Figure 3:**
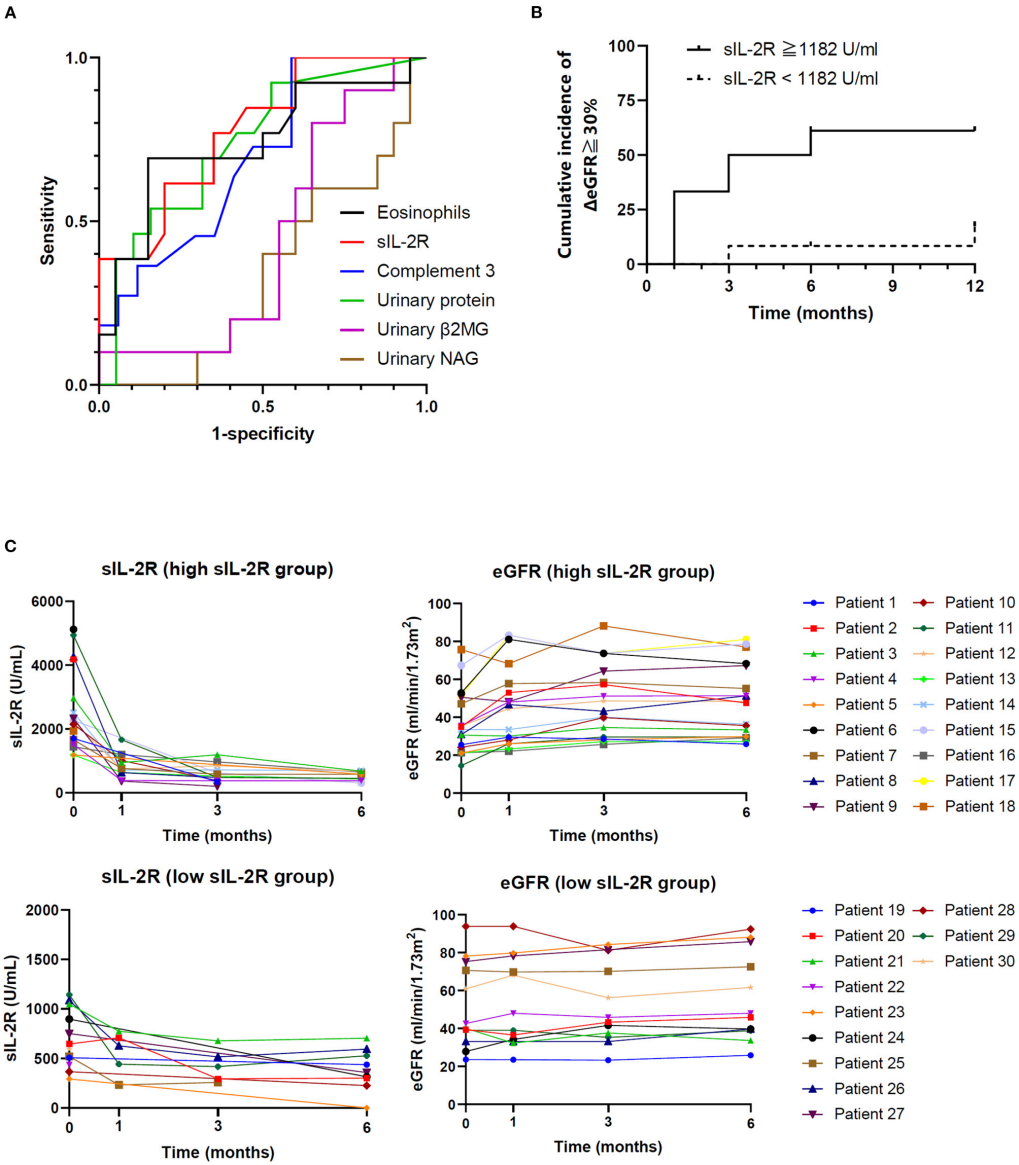
Predictive value of sIL-2R level for corticosteroid response. **(A)** Receiver operating characteristic curves of predictors of renal recovery (ΔeGFR ≥ 30%). **(B)** Cumulative incidence of renal recovery (ΔeGFR ≥ 30%) according to sIL-2R level. **(C)** Individual changes in eGFR and serum sIL-2R by the cutoff value of sIL-2R. TIN, tubulointerstitial nephritis; IgAN, immunoglobulin A nephropathy; DKD, diabetic kidney disease; sIL-2R, soluble interleukin-2 receptor; eGFR, estimated glomerular filtration rate; β2MG, beta2-microglobulin; NAG, N-acetyl-beta-D-glucosaminidase.

Receiver operating characteristic curve analysis was performed to determine the diagnostic reliability of relative variables for predicting renal recovery (defined as ≥30% increase in ΔeGFR). Urinary NAG and β2MG, which are urinary markers of tubular function, were also analyzed. Serum sIL-2R level (area under the curve [AUC] 0.805) was the most potent predictor of renal outcome, followed by complement 3 (AUC 0.754), bicarbonate (AUC 0.712), baseline eGFR (AUC 0.705), urinary β2MG (AUC 0.44), and urinary NAG (AUC 0.34) (Figure 3A). The clinical cutoff level with sensitivity ≥0.90 was 1,182 U/mL (sensitivity = 0.90, 1-specificity = 0.45), and the cumulative incidence of renal recovery in the high sIL-2R (≥1,182 U/mL) group (*n* = 18) was significantly higher than that in the low sIL-2R (<1,182 U/mL) group (*n* = 12) (*P* = 0.009) (Figure 3B). The individual patient responses to immunosuppressant within 6 months by the cutoff value of sIL-2R are shown in Figure 3C. Serum sIL-2R level was decreased after corticosteroid therapy with improvement of eGFR, especially in the high sIL-2R group. There were no significant differences between high sIL-2R and low sIL-2R group for any of baseline characteristics (data not shown).

## Discussion

This study assessed the clinical significance of sIL-2R in predicting disease outcome and therapeutic response to corticosteroids in patients with autoimmune TIN. Our findings demonstrated as follows: serum sIL-2R levels were significantly higher in patients with autoimmune TIN than those in IgAN and DKD patients with renal insufficiency in the similar extent; baseline sIL-2R in patients with autoimmune TIN was strongly correlated with renal recovery after corticosteroid therapy, and high sIL-2R (cutoff ≥1,182 U/mL) predicted the good therapeutic response.

Autoimmune TIN is diagnosed based on the clinical manifestations underlying the original disease and histological findings. However, these findings including rash, fever, urinalysis, histological interstitial fibrosis and infiltrates are not specific for TIN. In addition, TIN patients typically show elevated serum creatinine levels with no or mild urinary abnormalities. Dehydration-induced tubular injury and drug-induced nephrotoxicity also have similar characteristics in terms of clinical manifestation, but do not require immunosuppressive therapy. Thus, diagnosing TIN and assessing its disease activity is challenging for clinicians.

Several useful biomarkers, such as kidney injury molecule-1 (KIM-1), neutrophil gelatinase-associated lipocalin (NGAL), and monocyte chemoattractant protein-1 (MCP-1) have been identified as a predictor of acute kidney injury including TIN, however, it is difficult to distinguish TIN from other diseases with tubular injury using these markers (9, 10). Although urinary β2MG has been suggested as a diagnostic marker of TINU (11) and Balkan endemic nephropathy (12, 13), it is not specific to TIN. Recently, Moledina et al. (14) showed that urinary tumor necrosis factor-α and interleukin-9 help discriminate TIN from acute tubular injury. However, the relevance to prognosis and therapeutic response of these biomarkers were not evaluated. Li et al. reported that inflammatory makers including CRP and erythrocyte sedimentation rate in patients with TINU are not related to renal outcome by multivariate analysis (15).

Therefore, there is an emerged need for biomarkers to discriminate TIN patients who are appropriate for immunosuppressive therapy. Contradictory results have been reported on the availability of histological findings as a prognostic marker (16, 17). Muriithi et al. (18) reported that the severity of tubular atrophy and interstitial fibrosis was related to poor renal recovery. Moledina et al. (14) suggested that these histological findings are not always consistent with the clinical use because of inter-pathologist variability and sampling errors. In the underlying diseases of TIN including sarcoidosis (19, 20), IgG4-RKD (21), and Sjögren's syndrome (22), the higher serum sIL-2R level has been reportedly associated with the disease activity, although the association between sIL-2R and renal dysfunction was not evaluated. Elevated serum sIL-2R level have been reported in patients with active systemic lupus erythematosus (SLE) especially those with kidney involvement (23). Lundberg et al. (24) suggested that the plasma level of sIL-2Rα is predictive of renal disease progression in patients with IgAN. Therefore, we consider that serum sIL-2R in TIN should be assessed after excluding alternative diagnosis including SLE and IgAN. The clinical and histological findings make it possible to distinguish TIN from lupus nephritis/IgAN, because lupus nephritis / IgAN is characterized by urinary abnormalities with the presence of a variety of urinary casts and immune-mediated glomerular injury, whereas patients with TIN typically show no or mild urinary findings without glomerular lesion. In our study, elevated serum sIL-2R level, not histological findings, was an independent relative factor for therapeutic response in renal dysfunction. The serum sIL-2R may not be affected by irreversible fibrosis, and potentially predicting disease activity.

Numerous previous reports on TIN predominantly enrolled drug- and/or infection-induced TIN cases. Drug-induced TIN is implicated in allergic responses and requires the termination of offending drugs, and may necessitate immunosuppressive therapy. Autoimmune TIN shares some clinical features with drug-induced TIN, but evidence on autoimmune TIN is limited because of its rarity. In this study, high sIL-2R levels were positively correlated with renal recovery in response to corticosteroids. Elevated sIL-2R levels may be a potential predictive marker for therapeutic response in autoimmune TIN. This is the first study to report a candidate of biomarker of therapeutic response that might help assess disease activity and determine the therapeutic strategy in patients with autoimmune TIN.

This study had some limitations. First, this was a retrospective, single-center observational study with a limited sample size. Second, we did not analyze the natural course of TIN treated with only supportive therapy because of the small sample size. Third, we could not perform immunohistological evaluation of CD4 and CD8. Further prospective studies with a large number of samples will promise to establish relevance of serum sIL-2R in patients with autoimmune TIN in daily clinical practice as well as in designing clinical trials for the treatment of autoimmune TIN.

## Data Availability Statement

The raw data supporting the conclusions of this article will be made available by the authors, without undue reservation.

## Ethics Statement

The studies involving human participants were reviewed and approved by Ethical Review Board for Life Science and Medical Research, Hokkaido University Hospital. The patients/participants provided their written informed consent to participate in this study.

## Author Contributions

SS-A and DN: conceptualization, data curation, formal analysis, and writing the original draft. SN, YU, ME, AY, JYo, TK, KW-K, ST-O, JYa, NM, NK, FH, and TA: writing the original draft. SI, TT, and YF: resources. All authors have read and approved the final version of the manuscript.

## Conflict of Interest

The authors declare that the research was conducted in the absence of any commercial or financial relationships that could be construed as a potential conflict of interest.

## Publisher's Note

All claims expressed in this article are solely those of the authors and do not necessarily represent those of their affiliated organizations, or those of the publisher, the editors and the reviewers. Any product that may be evaluated in this article, or claim that may be made by its manufacturer, is not guaranteed or endorsed by the publisher.
