# Supplementary material for: Soluble Interleukin-2 Receptor Predicts Treatment Outcome in Patients With Autoimmune Tubulointerstitial Nephritis. A Preliminary Study
Source: Front Med (Lausanne). 2022 Feb 25;9:827388. doi: 10.3389/fmed.2022.827388 (PMC8914032; doi:10.3389/fmed.2022.827388)
Supplement: Supplementary file 1 [file Data_Sheet_1.doc]

**Supplementary Material**

**Supplementary Table 1: The characteristics of autoimmune TIN patients**

|  |  |  |  | **Treated** |  |  |  | **Untreated** |
| --- | --- | --- | --- | --- | --- | --- | --- | --- |
| **Characteristics** | **Total  (n = 30)** | **Sarcoidosis (n = 10)** | **TINU (n = 8)** | **IgG4-RKD (n = 6)** | **Idiopathic (n = 4)** | **Sjögren’s syndrome**  **(n = 1)** | **Castleman disease**  **(n = 1)** | **Total (n = 6)** |
| Age (year) | 61.0 (41.3-65.5) | 63.5 (55.5-69.0) | 36.5 (17.0-59.5) | 62.5 (53.8-69.3) | 45.5 (18.5-77.0) | 62 | 61 | 47.0 (28.5-67.5) |
| Male, n (%) | 15 (50.0) | 4 (40.0) | 4 (50.0) | 5 (83.3) | 2 (50.0) | 0 | 0 | 2 (33.3) |
| BMI (kg/m^2^) | 21.7±2.9 | 21.6±3.3 | 21.8±3.5 | 21.6±2.3 | 22.8±2.8 | 21.7 | 20.5 | 24.1±4.8 |
| Observation period  (months) | 36.0 (21.0-60.0) | 48.0 (10.5-60.0) | 48.0 (18.8-60.0) | 36.6 (30.0-60.0) | 42.2 (15.0-60.0) | 36.0 | 60 | - |
| **Clinical features** |  |  |  |  |  |  |  |  |
| Fever, n (%) | 1 (3.3) | 0 | 0 | 0 | 0 | 0 | 1 (100) | 0 |
| Skin rash, n (%) | 1 (3.3) | 1 (10.0) | 0 | 0 | 0 | 0 | 0 | 0 |
| Arthralgia, n (%) | 0 | 0 | 0 | 0 | 0 | 0 | 0 | 0 |
| Back pain, n (%) | 0 | 0 | 0 | 0 | 0 | 0 | 0 | 0 |
| **Blood tests** |  |  |  |  |  |  |  |  |
| White blood cells (/µL) | 5850  (4700-6600) | 5100  (3630-6600) | 5550  (4700-6300) | 6200  (5030-7730) | 6650  (5230-9950) | 3320 | 5900 | 5500  (3330-7880) |
| Eosinophils (/µL) | 179 (93-294) | 195 (112-323) | 175 (113-268) | 276 (193-591) | 129 (22-206) | 33 | 59 | 144 (74-384) |
| Hemoglobin (g/dL) | 11.7±1.4 | 12.2±1.2 | 11.8±1.2 | 11.7±1.5 | 11.3±1.2 | 9.7 | 8.5 | 11.8±2.3 |
| Platelets (10^4^/µL) | 24.1±5.0 | 24.4±6.5 | 25.1±3.4 | 22.3±3.5 | 25.4±7.3 | 21.4 | 21.3 | 26.2±8.7 |
| sIL-2R (U/mL) | 1480  (720-2280) | 2370  (1120-4430) | 840  (380-2130) | 1590  (800-2790) | 970  (570-1380) | 1180 | 1937 | 920  (530-2300) |
| Albumin (g/dL) | 4.00 (3.65-4.30) | 4.00 (3.90-4.20) | 4.15 (3.78-4.40) | 3.35 (2.85-4.35) | 4.25 (3.98-4.38) | 3.6 | 1.3 | 4.20 (3.55-4.60) |
| CRP (mg/dL) | 0.12 (0.05-0.61) | 0.12 (0.07-0.73) | 0.11 (0.01-0.22) | 0.19 (0.05-1.05) | 0.07 (0.01-0.59) | 0.33 | 20.21 | 0.02 (0-0.18) |
| pH | 7.38 (7.34-7.40) | 7.38 (7.34-7.39) | 7.38 (7.36-7.41) | 7.37 (7.36-7.40) | 7.35 (7.23-7.39) | 7.39 | 7.45 | 7.33 (7.28-7.38) |
| Bicarbonate (mEq/L) | 24.5±3.6 | 24.6±3.5 | 26.9±2.4 | 22.7±2.3 | 22.0±3.4 | 19.6 | 30.2 | 24.3±3.8 |
| Creatinine (mg/dL) | 1.31 (1.06-1.74) | 1.36 (1.25-1.75) | 1.12 (0.96-1.53) | 1.34 (0.96-1.71) | 2.07 (1.09-3.00) | 1.95 | 0.61 | 1.28 (0.85-1.55) |
| eGFR (mL/min/1.73m^2^) | 43.3±20.4 | 34.1±10.3 | 56.6±23.1 | 44.5±15.3 | 35.4±26.7 | 21.1 | 75.7 | 51.4±30.9 |
| BUN (mg/dL) | 19.0 (15.8-26.3) | 22.5 (16.5-26.0) | 16.0 (14.5-19.5) | 17.5 (14.0-23.5) | 30.0 (18.5-35.5) | 32 | 9 | 16.5 (10.5-20.0) |
| Immunoglobulin G  (mg/dL) | 1770  (1470-2340) | 1770  (1520-2050) | 1500  (1390-1710) | 2910  (2350-3890) | 1420  (1060-1780) | 3910 | 6360 | 1450  (1340-1880) |
| Complement 3 (mg/dL) | 94 (76-120) | 94 (90-108) | 106 (94-126) | 51 (41-71) | 117 (92-148) | 71 | 179 | 122 (81-159) |
| **Urinalysis** |  |  |  |  |  |  |  |  |
| Urinary protein (g/gCre) | 0.28 (0-0.43) | 0.07 (0-0.40) | 0.20 (0.01-0.37) | 0.31 (0-1.22) | 0.22 (0.04-0.61) | 0.49 | 3.96 | 0.22 (0-0.92) |
| Hematuria, n (%) | 7 (23.3) | 3 (30.0) | 2 (25.0) | 1 (16.7) | 0 | 0 | 1 (100) | 1 (16.7) |
| Leukocyturia, n (%) | 6 (20.0) | 3 (30.0) | 1 (12.5) | 2 (33.3) | 0 | 0 | 0 | 1 (16.7) |
| β2MG (mg/gCre) | 7.3 (1.0-22.3) | 4.9 (0.9-11.1) | 11.0 (0.7-28.1) | 4.2 (1.0-14.8) | 14.5 (4.9-68.9) | 46.2 | 264.7 | 18.1 (0.5-137.7) |
| NAG (U/gCre) | 14.6 (9.2-19.9) | 14.6 (11.6-16.9) | 7.6 (4.3-20.9) | 16.6 (10.5-23.2) | 11.4 (4.7-15.5) | 24 | 173.1 | 11.1 (6.6-43.2) |
| **Treatment** |  |  |  |  |  |  |  |  |
| Methylprednisolone pulse, n (%) | 4 (13.3) | 0 | 2 (25.0) | 0 | 2 (50.0) | 0 | 0 | - |
| Prednisolone dose  (mg/kg) | 0.56 (0.48-0.61) | 0.59 (0.56-0.69) | 0.45 (0.41-0.52) | 0.58 (0.55-0.63) | 0.49 (0.34-0.59) | 1.33 | 0.97 | - |
| **Pathology (n=17)** | 17 (56.7) | 7 (70.0) | 3 (37.5) | 5 (83.3) | 1 (25.0) | 1 (100) | 0 (0) |  |
| Inflammatory cells  infiltration to  interstitium (%) | 21.7  (17.7-47.1) | 21.3  (20.0-23.6) | 18.3  (10.0-32.5) | 56.7  (32.7-75.8) | 45.8 | 16 | - | - |
| Fibrosis (%) | 37.3±11.4 | 38.8±12.2 | 33.7±9.67 | 38.3±14.4 | 42.3 | 27.4 | - | - |

Values for categorical variables are given as number (percentage); values for continuous variables are given as means ± SDs (range) or medians (inter quartile ranges).

Abbreviations: TINU, tubulointerstitial nephritis with uveitis; IgG4-RKD, immunoglobulin G4 related disease; BMI, body mass index; sIL-2R, soluble interleukin-2 receptor; CRP, C-reactive protein;

eGFR, estimated glomerular filtration rate; BUN, blood urea nitrogen; Cre, creatinine; β2MG, beta2-microglobulin; NAG, N-acetyl-beta-D-glucosaminidase; TIN, tubulointerstitial nephritis.

**Supplementary Table 2: The characteristics of control group (IgA nephropathy and diabetic kidney disease).**

| **Characteristic** | **IgA nephropathy  (n = 17)** | **Diabetic kidney disease  (n = 9)** |
| --- | --- | --- |
| Age (year) | 49.0 (36.5-64.5) | 67.0 (61.5-70.5) |
| Male/female | 7/10 | 8/1 |
| BMI (kg/m^2^) | 22.9±3.8 | 26.9±5.0 |
| **Blood tests** |  |  |
| White blood cells (/µL) | 7300 (5400-8650) | 6200 (4800-7800) |
| Eosinophils (/µL) | 214 (118-409) | 187 (113-417) |
| Hemoglobin (g/dL) | 13.7±1.3 | 12.2±1.9 |
| Platelets (10^4^/µL) | 25.0±6.9 | 26.0±6.6 |
| sIL-2R (U/mL) | 530 (370-660) | 570 (480-720) |
| Albumin (g/dL) | 4.00 (3.90-4.30) | 4.10 (3.85-4.25) |
| CRP (mg/dL) | 0.03 (0-0.10) | 0.08 (0.03-0.18) |
| pH | 7.36 (7.34-7.40) | 7.32 (7.30-7.35) |
| Bicarbonate (mEq/L) | 22.7±1.5 | 23.1±2.5 |
| Creatinine (mg/dL) | 1.10 (0.77-1.51) | 2.07 (1.68-2.47) |
| eGFR (mL/min/1.73m^2^) | 57.8±36.2 | 27.7±9.1 |
| Blood urea nitrogen (mg/dL) | 21.0 (14.0-25.5) | 33.0 (30.0-37.5) |
| Immunoglobulin G (mg/dL) | 1160 (1010-1360) | 1200 (1170-1240) |
| Complement 3 (mg/dL) | 92 (80-102) | 91 |
| **Urinalysis** |  |  |
| Urinary protein (g/gCre) | 0.49 (0.16-1.02) | 1.88 (0.64-2.86) |
| Hematuria, n (%) | 13 (76.5) | 1 (11.1) |
| Leukocyturia | 1 (5.9) | 1 (11.1) |
| β2MG/Cr (mg/gCre) | 0.1 (0-2.8) | 0.1 (0-0.2) |
| NAG/Cr (U/gCre) | 10.2 (6.4-16.5) | 10.7 (9.9-11.4) |

Abbreviations: BMI, body mass index; sIL-2R, soluble interleukin-2 receptor; CRP, C-reactive protein; eGFR, estimated glomerular filtration rate; Cre, creatinine; β2MG, beta2-microglobulin; NAG, N-acetyl-beta-D-glucosaminidase.

**Supplementary Table 3: Correlations between sIL-2R at baseline and other baseline factors**

| **Variables** | **Correlation coefficient** | ***P* value** |
| --- | --- | --- |
| Age (year) | 0.069 | 0.715 |
| Sex | - | 0.469 |
| BMI (kg/m^2^) | 0.205 | 0.286 |
| Blood tests |  |  |
| White blood cells (/µL) | -0.203 | 0.281 |
| Eosinophils (/µL) | 0.194 | 0.305 |
| Hemoglobin (g/dL) | -0.063 | 0.74 |
| Platelets (10^4^/µL) | -0.223 | 0.236 |
| Albumin (g/dL) | -0.328 | 0.083 |
| CRP (mg/dL) | 0.298 | 0.11 |
| Bicarbonate (mEq/L) | -0.144 | 0.473 |
| eGFR (mL/min/1.73m^2^) | -0.286 | 0.126 |
| Immunoglobulin G (mg/dL) | 0.36 | 0.055 |
| Complement 3 (mg/dL) | -0.221 | 0.29 |
| Urinalysis |  |  |
| Urinary protein (g/gCre) | 9.316 | 0.124 |
| Hematuria | - | 0.327 |
| Leukocyturia | - | 0.074 |
| β2MG/Cre (mg/gCre) | 0.066 | 0.73 |
| NAG/Cre (U/gCre) | 0.241 | 0.199 |
| Pathology |  |  |
| Inflammatory cells infiltration  to interstitium (%) | 0.101 | 0.701 |
| Fibrosis (%) | -0.152 | 0.56 |

Abbreviations: BMI, body mass index; sIL-2R, soluble interleukin-2 receptor;

CRP, C-reactive protein; eGFR, estimated glomerular filtration rate; β2MG, beta2-microglobulin; NAG, N-acetyl-beta-D-glucosaminidase.
